# Nebulized perflubron and carbon dioxide rapidly dilate constricted airways in an ovine model of allergic asthma

**DOI:** 10.1186/s12931-014-0098-x

**Published:** 2014-09-16

**Authors:** Tamer Y El Mays, Parichita Choudhury, Richard Leigh, Emmanuel Koumoundouros, Joanne Van der Velden, Grishma Shrestha, Cora A Pieron, John H Dennis, Francis HY Green, Ken J Snibson

**Affiliations:** Airway Inflammation Research Group, Snyder Institute for Chronic Diseases, University of Calgary, Calgary, AB Canada; Centre for Animal Biotechnology, University of Melbourne, Melbourne, VIC Australia; Lung Health Research Centre, University of Melbourne, Melbourne, VIC Australia; SolAero Ltd, Calgary, AB Canada; Department of Pathology & Laboratory Medicine, HRIC 4AC62 3280 Hospital Drive N.W, Calgary, AB T2N 4Z6 Canada

**Keywords:** Asthma, Novel treatments, Sheep model, Allergic airway disease, Carbon dioxide, Perflubron

## Abstract

**Background:**

The low toxicity of perfluorocarbons (PFCs), their high affinity for respiratory gases and their compatibility with lung surfactant have made them useful candidates for treating respiratory diseases such as adult respiratory distress syndrome. We report results for treating acute allergic and non-allergic bronchoconstriction in sheep using S-1226 (a gas mixture containing carbon dioxide and small volumes of nebulized perflubron). The carbon dioxide, which is highly soluble in perflubron, was used to relax airway smooth muscle.

**Methods:**

Sheep previously sensitized to house dust mite (HDM) were challenged with HDM aerosols to induce early asthmatic responses. At the maximal responses (characterised by an increase in lung resistance), the sheep were either not treated or treated with one of the following; nebulized S-1226 (perflubron + 12% CO_2_), nebulized perflubron + medical air, 12% CO_2_, salbutamol or medical air. Lung resistance was monitored for up to 20 minutes after cessation of treatment.

In additional naïve sheep, a segmental bronchus was pre-contracted with methacholine (MCh) and treated with nebulized S-1226 administered via a bronchoscope catheter. Subsequent bronchodilatation was monitored by real time digital video recording.

**Results:**

Treatment with S-1226 for 2 minutes following HDM challenge resulted in a more rapid, more profound and more prolonged decline in lung resistance compared with the other treatment interventions. Video bronchoscopy showed an immediate and complete (within 5 seconds) re-opening of MCh-constricted airways following treatment with S-1226.

**Conclusions:**

S-1226 is a potent and rapid formulation for re-opening constricted airways. Its mechanism(s) of action are unknown. The formulation has potential as a rescue treatment for acute severe asthma.

**Electronic supplementary material:**

The online version of this article (doi:10.1186/s12931-014-0098-x) contains supplementary material, which is available to authorized users.

## Background

Asthma is a common chronic disorder characterized by airway hyper-responsiveness, bronchial smooth muscle hypertrophy, increased mucus secretion, and airway inflammation [1,2] on a background of airway wall remodelling and mucous plugs [3,4]. Asthma is triggered by many factors such as allergens (e.g., pollen, pet dander), irritants (e.g., smoke), drugs, cold, exercise [5], and upper respiratory tract infections [6]. Based on recent trends, the worldwide prevalence of asthma may increase from 300 million individuals currently to 400 million individuals by 2025 [7]. Healthcare costs of asthma are considered to be among the highest associated with chronic disease [8,9]. In Canada, 59% of asthma patients treated in general practice are estimated to have uncontrolled disease [10]. Inhaled short acting β_2_-agonists represent the most common front-line emergency department treatment for acute exacerbations due to their well-known rapid bronchodilatory effect [11–13] β_2_-agonists, while generally considered safe may have significant side effects [14] and approximately one fifth to one third of patients do not respond to front-line treatment with short acting β_2_-agonists and ultimately require hospitalisation [15]. Furthermore, a clinically significant proportion of asthma patients are refractory to conventional treatments, posing a challenge for their management [16]. A new class of bronchodilators working through a different mechanism would be a useful addition for treating acute asthma.

In acute severe asthma, conventional treatment with inhaled β_2_ agonists may no longer be effective [11–13], as the therapeutic aerosol may be unable to penetrate airways obstructed by mucous plugs [4], increasing the risk of sudden death [17]. Emergency treatment for asthma includes mechanical ventilation [15] which may cause injury (barotrauma) to the ventilated segments of the lung [18]. Hence, a rapid non-invasive treatment for severe, life-threatening asthma is needed.

Inhaled carbon dioxide is a known bronchodilator for many mammalian species [19]. Hypercapnia has long been known to relax airway smooth muscle in vitro [20,21], whereas hypocapnia causes airway smooth muscle contraction [22]. Hyperventilation is a well known trigger of asthma [23]. Studies by Fisher et al. [24] and Mc Fadden et al. [25] indicate that low alveolar PCO_2_ (PACO_2_) may be associated with an increase in airway tone in asthmatic patients. By contrast hypercapnia (induced by a short circuiting CO_2_ absorber) has been shown to reduce airway resistance in both healthy and asthmatic subjects [26]. Finally a study reported by Fisher and Hansen [27] showed that inhalation of 6% CO_2_ over 4–5 minutes relieved exercise induced airflow obstruction in young atopic asthmatics at rest and during exercise.

Perfluorooctylbromide (perflubron) is a chemically inert perfluorocarbon (PFC). It has a high solubility for respiratory gases such as CO_2_ and O_2_ and a low surface tension compatible with endogenous airway surfactant [28–30]. It has been used in total and partial liquid ventilation studies for treatment of ARDS [28]. In addition, perflubron has been shown to reduce pulmonary inflammation and injury through a variety of actions [28,31–33]. The high density and low surface tension of perflubron should also facilitate the penetration of obstructed airways and re-expansion of collapsed lung [34].

There are no reported studies of PFC use in asthma. However, its properties of gas transport for CO_2_ and O_2_, low interfacial tension at the PFC-mucus interface, low toxicity and anti-inflammatory properties [31–33,35] make it a good candidate as a rescue therapy in life threatening acute asthma. Recently perflubron aerosols have been used to successfully treat acute lung injury in an animal model [36,37].

The major challenge for treating acute severe asthma is to open constricted airways rapidly enough to allow delivery of conventional medication to the diseased airways.

In this study we show that inhaled nebulized perflubron + CO_2_ (S-1226) rapidly opens constricted airways in an ovine model of allergic asthma using house dust mite as allergen [38,39].

## Methods

### Experimental sheep

Female merino-cross farm-reared sheep aged 6 months were used in the experiments described here. After the sensitisation procedures (described below), sheep were transported to Animal House Facility at Veterinary Science, Parkville, Australia where they were kept in indoor pens for all the procedures described in this report. On arrival at the Animal House Facility all sheep were treated with an antihelminthic to ensure that they were parasite-free.

All experimental protocols used in this study were approved by the Animal Experimentation Ethics Committee at the University of Melbourne.

### Drug source

Acetyl-β-methylcholine chloride (MCh) was from Sigma-Aldrich, Stenhein, Germany and salbutamol sulphate solution was purchased from GSK Pty Ltd Australia. Gas mixture (12% CO_2_ + 21% O_2_+ balance N_2_) was from CIG Pty Ltd Australia.

### Characterization of nebulized perflubron

Characterization of perflubron delivered by the Sidestream nebulizer was conducted under cGMP conditions at SolAero Ltd laboratory. Methods to characterize perflubron aerosol were adapted from regulatory methods embodied in the European Nebulizer Standard [40]. The unique nature of perflubron required adaptation of methods including incorporation of trace quantities (5%) of a perflubron compatible non-volatile PFC which could be assayed gravimetrically to determine both aerosol output as well as particle size distributions. Aerosol output was determined using a breath simulation (500 mL at 15 cycles per minute) and collecting released aerosol representing the ‘inhaled aerosol’ on a pre-weighed 3M electrostatic filter, subsequently allowing volatile perflubron to dissipate and quantitatively weighing the non-volatile PFC component. The residual weight gain was then used to back calculate the original amount of deposited aerosol. Pre- and Post- weights of nebulizers provided the gross perflubron emitted, from which the analyzed aerosol proportion could be subtracted to estimate the proportion of perflubron in the vapor phase. Aerosol size was determined using a Marple 298X cascade impactor equipped with pre-weighed GF/A filters from which the non-volatile aerosol residue was similarly gravimetrically determined to quantify size fractions.

### House dust mite (HDM) sensitization and airway challenges

Sheep 6 months of age were subcutaneously sensitized with 50 μg house dust mite (HDM) extract (Dermatophagoides pteronyssinus; CSL Ltd, Parkville, VIC, Australia) mixed 1:1 in aluminum hydroxide [Al(OH)_3_] adjuvant. Injections of 1 ml HDM / Al(OH)_3_ were given on three occasions at 2 week intervals [38]. Levels of HDM-specific IgE (IgE-HDM) were determined by enzyme-linked immunosorbent assay (ELISA) of serum samples obtained from animals immediately prior to, and 7 days after, HDM sensitization [38,41]. Sheep were only selected for use in the study if they had increased levels of HDM-specific IgE (IgE-HDM) ≥2-fold above pre-sensitization values.

To induce pulmonary sensitization to HDM, all sheep were given three inhalation challenges of aerosolised HDM (1 mg/ml in 5 ml of phosphate buffered saline). Lung function was recorded for one hour directly after the aerosol HDM challenge. HDM aerosols were generated with a medical nebulizer (Vitalair RapidNeb, Allersearch, Vermont, Australia). The nebulizer was connected to a dosimeter system consisting of a solenoid valve and a source of compressed air (20 psi). The output of the nebulizers was directed into a T-piece with one arm connected to the inspiratory port of a mechanical ventilator (Intermed Bear 2 – Adult Volume Ventilator, Bear Medical Systems, Inc., Riverside, CA). HDM challenges were delivered at a tidal volume of 500 mL and a rate of 20 breaths per minute for 10 minutes.

### Measurement of lung function

Data on lung function were obtained from all sheep using established techniques [42]. Briefly, pressures and flow were acquired from conscious unsedated sheep using a National Instruments Data Acquisition (DAQ) system with a Virtual Instrument programmed with Lab View® (National Instruments, Austin, USA). The sheep were restrained in a body sheath and head restraining harness tethered to a custom-made animal trolley. To measure oesophageal pressures, a balloon pressure catheter was advanced through one nostril into the lower esophagus. To assess tracheal pressures throughout the respiratory cycle a cuffed endotracheal tube was placed into the trachea and then a side-hole catheter was advanced 2 cm past the distal end of the endotracheal tube. The oesophageal and tracheal catheters were connected to differential transducers (Gaeltec 8 T, Gaeltec Ltd, Dunvegan, Scotland) to measure oesophageal pressure (P_oes_) and airway pressures (P_aw_) respectively. Transpulmonary pressure (P_l_) was obtained from P_oes_ with respect to P_aw_. Airflow was measured via a pneumotachograph (Hans Rudolph Inc, Kansas City, USA) attached to the proximal end of the endotracheal tube. Calibration and synchronization of the gas transducers and pneumotachograph were performed as described previously [43,44]. Lung dynamics were determined by a modified Mead-Whittenberger method [45]. Breath by breath analysis of flow and pleural pressure (P_pl_) was used to determine lung resistance (R_L_) from an average of five breaths. Data from breaths which differed by more than 20% in volume and more than 10% of the breathing rate were rejected. Acquisition and detailed analysis of pulmonary physiological data were performed on customized Lab View software developed by Koumoundouros et al. [42].

### Treatment delivery

Treatments with nebulized S-1226, nebulized perflubron and nebulized salbutamol were generated with a Sidestream® nebulizer (Philips Respironics). The nebulizer was connected to a dosimeter system consisting of a solenoid valve and a source of compressed (20 psi driving pressure) gas mixture (12% CO_2_, 21% O_2_ and 67% N_2_) for S-1226 or compressed medical air (21% O_2_ and 79% N_2_) for perflubron and salbutamol. Treatments were delivered at a tidal volume of 500 mL and a rate of 20 breaths per minute for 2 minutes. Treatments with CO_2_ alone and medical air were performed following the same protocol but with an empty nebulizer.

### HDM challenge followed by treatment

Sheep (previously HDM sensitized) were challenged with HDM aerosols to induce early phase asthmatic responses. At the peak of these responses (characterised by an increase in lung resistance), the sheep were treated for two minutes with one of six treatment groups listed below. Lung resistance was monitored up to 20 minutes following cessation of treatment.

1. S-1226: Nebulized perfluorooctylbromide (perflubron) generated using a Sidestream® nebulizer (Philips Respironics) and driven by a compressed gas mixture containing 12% CO_2_, 21% O_2_ and balance N_2_.
2. CO_2_ alone: Inhalation of a gas mixture containing 12% CO_2_, 21% O_2_ and balance N_2_.
3. Perflubron: Nebulized perflubron driven by compressed medical air.
4. Salbutamol (1 mg/ml): Nebulized salbutamol driven by compressed medical air.
5. Medical air: Inhalation of medical air
6. No treatment: room air

### Video bronchoscopy

An unsensitised sheep was used in separate experiments to visually assess the airway response to inhaled S-1226. Real time video recordings were taken with a bronchoscope digital camera following methacholine challenge. To insure that the same airway was recorded throughout the procedure, the upper region of the airway was tattooed by injecting tissue dye using a transbronchial needle. The airway was then pre-contracted with an aerosol of methacholine (MCh) delivered via a Trudell Ltd Pty bronchoscope catheter, inserted into the biopsy port of the bronchoscope. Doubling doses of methacholine were nebulized directly into the lung segment (0.009, 0.018, 0.037 w/v) for 30 seconds with a 2 minute interval between methacholine doses until the airway was maximally constricted. The airway was then treated with nebulized S-1226 delivered through a Trudell Pty Ltd bronchoscope catheter and the effects video recorded in real time. If the airway re-constricted, second and third treatments were administered.

### Data analysis

In each experiment several intervals were analysed: Baseline, HDM-induced early phase response, and three post-treatment periods (immediately, 1–10 minute and 10 to 20 minutes after treatment cessation). Means were calculated for each interval and treatment effect was expressed as percent decline of lung resistance.

Analysis of variance (ANOVA) was performed over each time interval to determine if any of the treatment groups performed better than any other. A two tailed Dunnett’s post-hoc test was performed. This involved separate analyses addressing two distinct questions. The first: is S-1226 superior to no treatment, medical air (negative control) or salbutamol (positive control). Secondly to determine which component of S-1226, CO_2_ or perflubron contributed greater to its efficacy. Tests were performed using GraphPad Prism version 5.00 and R version 3.1.1 for windows. A p-value ≤ 0.05 was considered significant.

## Results and discussion

### Characterization of nebulized perflubron

**Figure 1:**
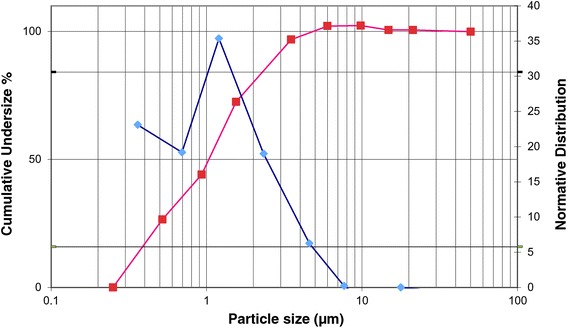
**Sidestream nebulizer aerosol size distribution.** Aerosol size is on the X-axis is plotted against the normative and cumulative underside aerosol distributions. The blue boxes profile the normative size distribution of nebulized perflubron, the near symmetry suggests a log-normal distribution of aerosol particles. The red squares plot the same data but as a cumulative size distribution of particle size against cumulative undersize %. The cumulative size distribution is used to interpolate the MMAD as the particle size at 50% of the cumulative mass – in this case the MMAD is 1.1 um. The Geometric Standard Deviation (GSD) of the MMAD is calculated using the intercepts at 15.8% and 84.1% (approximated by grey lines in Figure) using the formula GSD = sq rt (size 84.1um / size 15.8 um).

Results from characterization of nebulized perflubron are summarized in Table 1. The mean rate of perflubron delivered was 598 mg/minute, of which some 203 mg/minute consisted of aerosol with the remaining 395 mg/minute was estimated to be in vapor form – thus the majority (approximately 2/3 of the nebulized perflubron) was delivered in vapor form. The perflubron which was delivered in aerosol form demonstrated a relatively small particle size distribution, with a Mass Median Aerodynamic Diameter (MMAD) of 1.1 um, with a Geometric Standard Deviation (GSD) of 2.5 (Table 1). The perflubron aerosol size distributions obtained from the Sidestream nebulizer are shown in Figure 1.

**Table 1:** **Characterization of nebulized perflubron using the Sidestream (venturi closed)**

		**Nebulized perflubron**			**Aerosol size**	
**Sidestream** ***venturi CLOSED***	**Nebulizer flow rate L/min**	**Inhaled perflubron aerosol 1 minute mg**	**Inhaled perflubron Vapor 1 minute mg**	**Inhaled Total perflubron aerosol + vapor 1 minute mg/min**	**MMAD μm**	**GSD**
Exp#1	8.5	208	392	600	1.1	2.4
Exp#2	8.5	198	399	597	1.1	2.6

Nebulized perflubron was characterized in two separate experiments (exp#1 and exp#2) using compressed gas flow rate of 8.5 L/min through a Sidestream nebulizer with active venturi closed. Nebulized perflubron liquid was determined to partition into aerosol and vapour states, with the vapour being the predominant physical form (means: 395 mg/min vapour vs 203 mg/min aerosol). The droplet size of the aerosolized perflubron component was further examined and fractionated using a Marple 298X cascade impactor. The nebulized perflubron aerosol distribution curve showed near log normal symmetry (see normative distribution, Figure 4) having a Mass Median Aerodynamic Diameter (MMAD) of 1.1 um, and a Geometric Standard Deviation (GSD) of 2.5.

The characterization of nebulized perflubron demonstrated that the majority of the nebulized perflubron existed in the vapor form while the remaining proportion of aerosol existed as a relatively fine aerosol with an MMAD of 1.1 um. We infer that perflubron as vapor and aerosol, as well as CO_2_ gas component of inhaled S-1226, would rapidly distribute throughout the respiratory tract.

### HDM challenge followed by treatment

**Figure 2:**
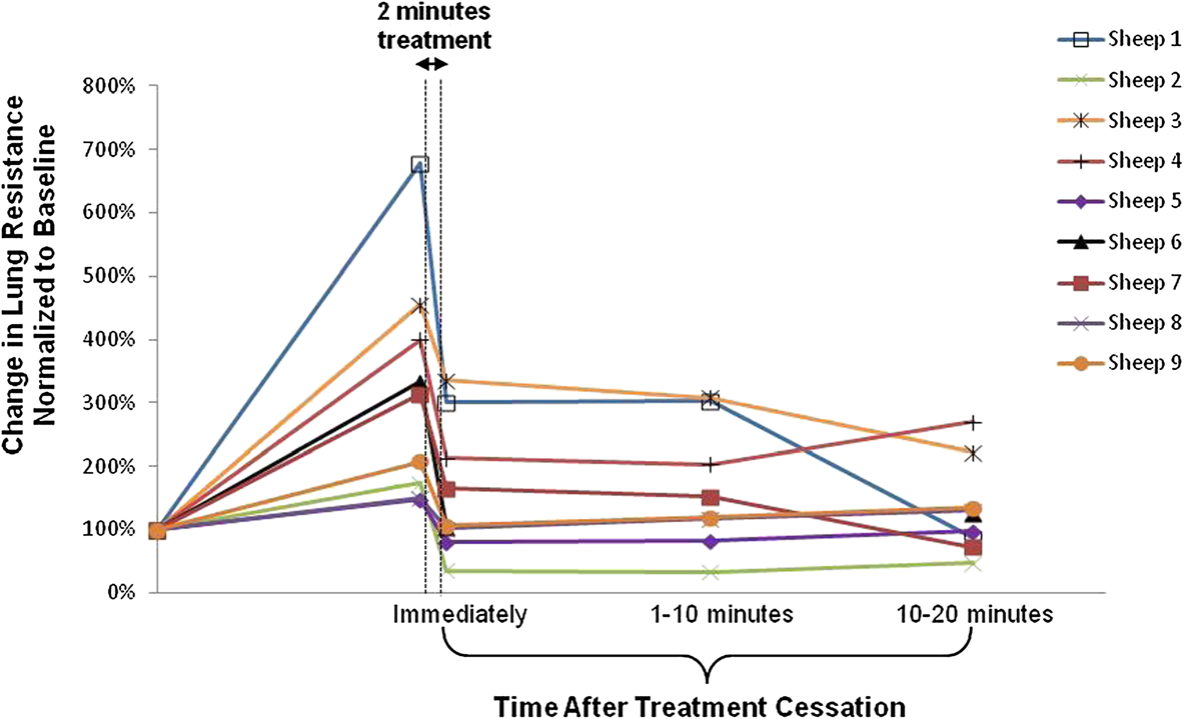
**Effect of S-1226 on lung resistance in the sheep model of asthma.** Changes in lung resistance following treatment with S-1226 in 9 different sheep. The treatment was delivered for 2 minutes and lung resistance was measured immediately, and at 1–10 minutes and 10–20 minutes after treatment cessation. All nine sheep showed an immediate and sustained decline in lung resistance following S-1226 treatment given at the peak of the early phase response to inhaled allergen (HDM).

Sensitized and HDM challenged sheep treated with S-1226 for 2 minutes during the peak of the early phase allergic response showed an immediate decline in lung resistance in all animals following HDM challenge (Figure 2).

**Figure 3:**
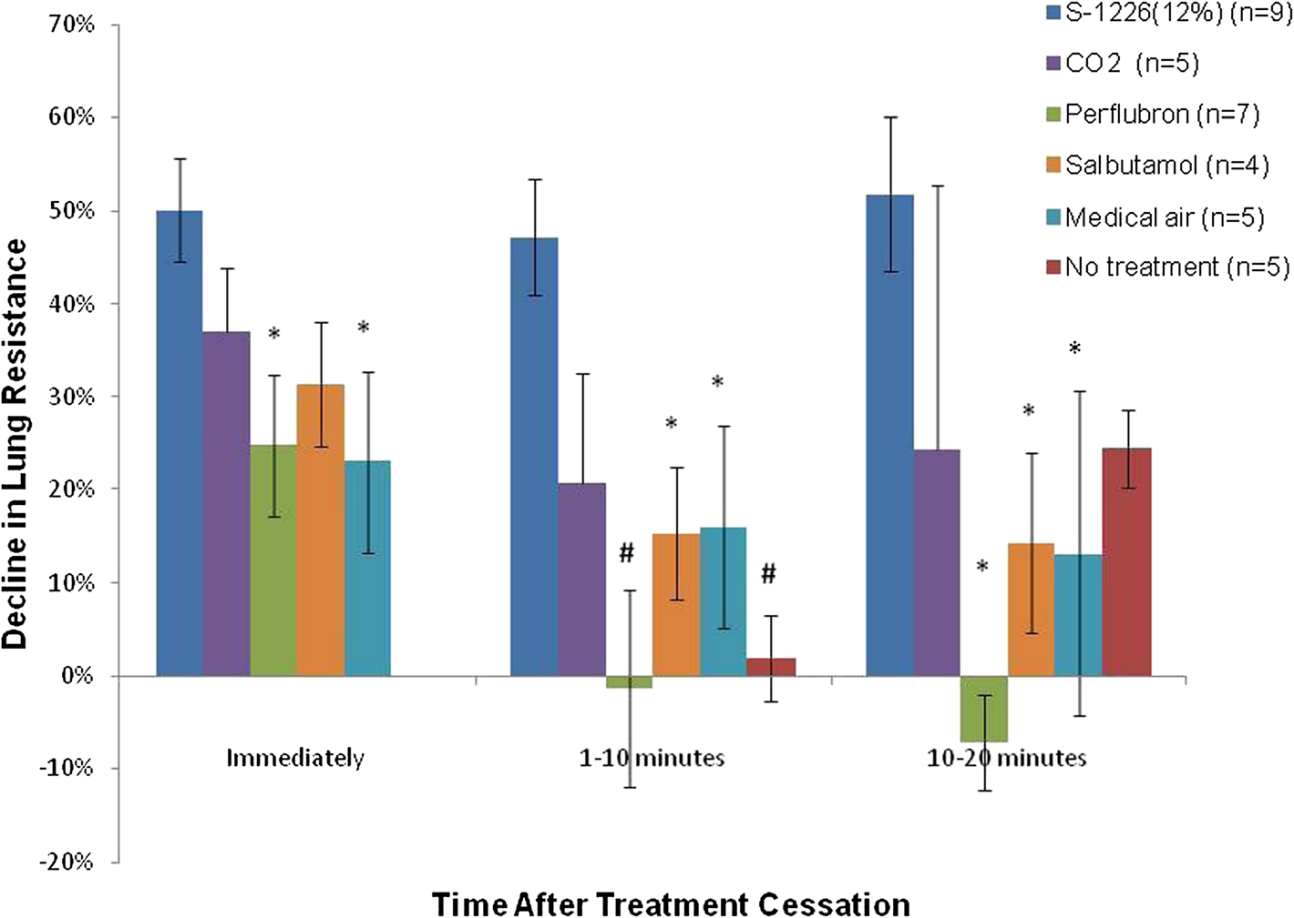
**Comparison between S-1226 and other bronchodilators in the sheep model of asthma.** This figure shows the % decline in lung resistance following treatment with S-1226, 12% CO_2_, perflubron, salbutamol, medical air or no treatment in HDM challenged sheep. Measurements were taken immediately, 1–10 minutes and 10–20 minutes after treatment. Treatment with S-1226 showed significant and sustained declines in lung resistance compared to medical air or no treatment. In addition S-1226 showed a significantly greater decline in lung resistance compared to salbutamol. P-values show significant difference from S-1226; *p <0.05, # p ≤ 0.01.

Treatment with S-1226 compared to the other arms of the study are shown in Figure 3. Lung resistance declined by 50.1% ± 5.5, 47.2% ± 6.2 and 51.8% ± 8.3 (mean ± SE) immediately, 1 to 10 minutes and 10 to 20 minutes after treatment with S-1226 respectively. Treatment with 12% CO_2_ resulted in declines in lung resistance of 36.9% ± 6.8, 20.6% ± 12.0 and 24.3% ± 28.4 (mean ± SE) immediately, 1 to 10 minutes and 10 to 20 minutes after treatment respectively. Treatment with perflubron + medical air resulted in declines in lung resistance of 24.8% ± 7.5, −1.3% ± 10.7 and −7.2% ± 5.1 (mean ± SE) immediately, 1 to 10 minutes and 10 to 20 minutes after treatment respectively. Treatment with salbutamol resulted in declines in lung resistance of 31.3% ± 6.8, 15.3% ± 7.1 and 14.3% ± 9.6 (mean ± SE) immediately, 1 to 10 minutes and 10 to 20 minutes after treatment respectively. Following inhalation of medical air, lung resistance declined by 23.1% ± 9.7, 16.0% ± 10.8 and 13.1% ± 17.5 (mean ± SE) immediately, 1 to 10 minutes and 10 to 20 minutes after treatment respectively. Lung resistance measured following HDM challenge with no treatment decreased by 1.9% ± 4.7, 24.4% ± 4.2 (mean ± SE) at 1 to 10 minutes and 10 to 20 minutes after treatment respectively.

Inhalation of nebulized S-1226 caused a rapid and sustained decrease in lung resistance during the early phase asthmatic response which was significantly greater than no treatment at 1–10 minutes (*p* = 0.002) and significantly greater than medical air immediately, 1–10 minutes and 10–20 minutes after treatment (*p* = 0.027, 0.018 and 0.039 respectively). The effect of S-1226 was significantly greater than salbutamol 1–10 minutes and 10–20 minutes after treatment (*p* = 0.025 and 0.046 respectively). S-1226 showed a significantly greater decline in lung resistance than perflubron alone immediately, 1–10 minutes and 10–20 minutes after treatment (*p* = 0.019, 0.002 and 0.012 respectively). Finally, the effect of S-1226 was stronger but not significantly different from CO_2_ alone immediately, 1–10 minutes and 10–20 minutes after treatment (*p* = 0.329, 0.124 and 0.319 respectively). The CO_2_ decline in lung resistance appeared shorter-lasting than the decline observed with S-1226.

### Video-bronchoscopy of S-1226 treatment following MCh challenge

**Figure 4:**
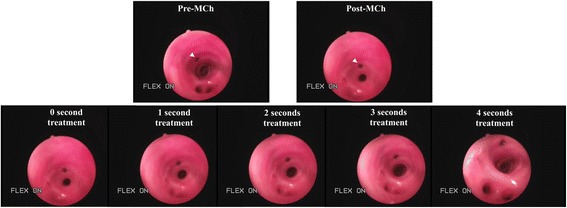
**Video bronchoscopy of the effect of S-1226.** Bronchoscopic still images of sheep airways pre and post MCh and seconds after initiating treatment with nebulized S-1226 (arrow heads indicate tissue dye). The video presentation is available online under Additional file 1.

The rapidity of the effect was demonstrated by video-bronchoscopy. MCh challenge resulted in rapid constriction of the segmental and subsegmental bronchi. Without treatment the airway narrowing lasted for at least 20 minutes before a slow natural recovery. Following treatment with a spray of S-1226 there was an immediate re-opening (within 5 seconds) of the MCh-constricted airway (Figure 4). A video recording of this treatment effect can be viewed online under Additional file 1. The effect was sustained during the delivery of S-1226 and up to 1 minute after treatment cessation. After that the airway began to re-constrict. Second and third treatments with S-1226 had identical effects.

### Potential mechanisms of action

Our study did not attempt to determine the mechanism of action of S-1226. The bronchodilatory effect of CO_2_ has been demonstrated in many mammalian species including cats, rats dogs [19,22] as well as sheep and humans However, the literature indicates that it might operate through several distinct mechanisms.

The first mechanism is through a direct physiologic relaxant effect of CO_2_ on airway smooth muscle [20–22]. The way(s) in which CO_2_ regulates bronchial smooth muscle tone is not fully understood. Recent *in vitro* data indicates that part of this effect is mediated by pH dependent and part by pH independent but epithelium dependent mechanisms [22]. Studies in vivo indicate the response (in rats) to inhaled CO_2_ does not require adrenergic pathways [46]. Fisher and Hansen [27] showed in their study of exercise induced asthma that cholinergic pathways were not involved in the bronchodilatory effects of inhaled CO_2_. Bronchodilator drugs that use β- adrenergic and cholinergic pathways form the current basis for treating obstructive lung disease.

Inhaled carbon dioxide (5, 10 and 15%) has been shown to reduce total lung resistance induced by intravenous histamine, acetylcholine and serotonin in dogs, cats and rats [19]. Inhaled CO_2_ has also been shown to reduce total lung resistance induced by pulmonary artery occlusion in cats and dogs [19]. All species studied responded to CO_2_ but there were small differences in response to the different broncho-constricting agents between species.

Studies by Ingram and colleagues [47] suggest that the bronchodilatory effect of CO_2_ may be independent of blood PCO_2_. They studied the effect of varying airway and arterial PCO_2_ on airway tone in dogs. They showed that low airway PCO_2_ combined with high arterial PCO_2_ resulted in a large increase in airway resistance [47]. These findings indicate that CO_2_ may have unique properties when delivered through the airways and supports the previously cited in vitro work that airway epithelium plays a part in the relaxant response [22]. Finally the rapidity of the response as seen in the sheep video indicates that neural mechanisms are involved. The non-adrenergic-non cholinergic (NANC) pathways are the best candidates for this effect. The NANC system has both broncho-constrictive and bronchodilator components [48]. CO_2_ alone or S-1226 may activate the bronchodilatory pathways or inhibit the broncho-constrictive pathways. Further work is required to address these questions.

The second mechanism may enhance delivery of CO_2_ to the airways. It could do this in several ways; first sustained release of CO_2_ dissolved in perflubron droplets as the droplets evaporate may explain the temporal synergism observed with the combination treatment. Second, because much of S-1226 is in gaseous form, it will be delivered rapidly into obstructed airways by diffusion and possibly through collateral ventilation.

The third mechanism whereby this combination treatment may work is by facilitating penetration and lubrication of mucus plugs, thus enhancing muco-ciliary clearance. We recently reported, using an in vitro system, that mucin plug clearance was significantly enhanced by perflubron [49].

S-1226 may also lower the surface tension in the inflamed airways. It has been shown that the normal airway is lined by a thin film of endogenous surfactant with a low surface tension [50]. This film is important for maintaining small airway patency at low lung volumes [51] and is abnormal in asthma [52,53].

Finally, carbon dioxide and perfluorocarbons may also be beneficial for their anti-inflammatory properties. Perflubron given in vitro or during partial liquid ventilation exerts an anti-inflammatory effect on alveolar cells [54], decreases white blood cell counts, [54,55] and the pro-inflammatory cytokines IL-1 and IL-6. Perflubron also increases release of the anti-inflammatory chemokine IL-10 [54].

### Strengths and limitations of the animal model

Like with most experimental systems, large animal models of allergic airway disease have both strengths, and also limitations, which need to be recognised when evaluating new therapies for asthma [56–58]. For example, clinically efficacious anti-asthma drugs such as leukotriene LTE4 receptor antagonists, cromolyn and corticosteroids have all been successfully tested in the sheep model of asthma [59]. The success of these drugs is in contrast to the testing of platelet activated factor (PAF) antagonist therapies in sheep. PAF antagonists were shown to alleviate allergic airway responses in sheep [60], however the positive outcome with these treatments in sheep did not translate to humans giving negative results in clinical trials [61]. Nevertheless, sheep are an appropriate species to investigate the efficacy of the bronchodilator reported here for a number of important reasons. Firstly, the sheep respiratory system is at least comparable to the human system with respect to lung size, lung anatomy and morphology, and the pharmacological and physiological parameters of the ovine lung [57–59]. Pertinent to this study, as the mechanism of the bronchodilator most likely involves both the airway smooth muscle and the intact airway epithelium [22], is that the arrangement and morphology of ovine airway epithelium [62] and airway smooth muscle down the tracheo-bronchial tree [57] is functionally similar to that observed in human lungs. Thus, the bronchodilator interactions we report here for the sheep model could reasonably translate to the human lung. The other major asset of the sheep model for examining bronchodilator effectiveness relates to the docile nature of sheep, which allows experiments to be ethically conducted in unsedated and unanaesthetised animals [59,63]. These anaesthetic and/or sedation agents, if administered as is necessary for behavioural reasons in most other animal species modeling asthma, may otherwise dampen, and thus mask, the bronchodilator responses.

In summary, we provide evidence that treatment with inhaled S-1226 delivered as an aerosol/gas mixture may play an important role in the treatment of acute asthma attacks. We show that perflubron combined with 12% CO_2_ is a potent airway smooth muscle relaxant in this ovine model of allergic asthma. The response to the combination treatment was rapid and exceeded treatment with medical air (negative control) and salbutamol (positive control) in both amplitude and temporal components. Further work is required to fully evaluate the mechanism(s) of action of this novel therapy.

## Additional file

Additional file 1:
***Video clip description:*** **the black dot is the stain from the tissue dye.** The partially occluded airway that is in the center of the screen begins to dilate immediately after the treatment starts (with the click sound on the video). Note that the adjacent smaller, completely occluded, airways also re-opened with treatment.
